# Lithotripsy, as Applied to the Destruction of Small Vesical Calculi

**Published:** 1872-11

**Authors:** J. W. S. Gouley

**Affiliations:** Surgeon to Bellevue Hospital, New York


ATLANTA

### MEDICAL AND SURGICAL JOURNAL.

VOL. X.—NOVEMBER, 1872.—NO. 8.

---

### ORIGINAL COMMUNICATIONS.

---

### LITHOTRIPSY AS APPLIED TO THE DESTRUCTION OF SMALL VESICAL CALCULI:

*WITH TWO ILLUSTRATIVE CASES.*

BY J. W. S. GOULEY,

*Surgeon to Bellevue Hospital, New York.*

The modern operation of lithotripsy—the reduction of vesical calculi to powder, or to a coarsely granular state, without the use of the knife—is indicated in cases where the stone is free and of medium or small size; where the general condition of the patient is good, his bladder tolerates instruments, and his urethra is capacious, or can be rendered so without risk to his life. I have said *modern operation*, because lithotripsy of the last seven or eight years is almost a new creation. When the straight and even the first curved instruments were in use, lithotripsy was a formidable and often serious undertaking; but now, with our simple devices, the operation often lasts only two or three minutes, instead of half an hour or more, as in the old way, and the introduction of the instrument is effected with as much ease and as little pain as the passage of a heavy steel sound of short curve.

Quite as much skill is required to detect a small stone in the the bladder as to destroy it, and we should all cultivate great care, gentleness and patience in our explorations, which should be aided with properly constructed instruments—the best being, I believe, Thompson's hollow exploring sound, with a short bulbous beak, an eye one inch from the point, and a stopper at the distal extremity, that the urine may be drawn off, or water injected, if it be desirable to increase the quantity of fluid in the bladder. Some prefer to use a light lithotriptor, by which the size of the stone can be ascertained at once.

It is a lamentable fact that some surgeons, who possess the required skill to find small stones, cut for, rather than crush them. I have, on many occasions, witnessed the performance of lithotomy for soft calculi not exceeding half an inch in diameter, where the other indications for lithotripsy were clear, and a successful result might have been anticipated from a simple crushing. It seems to me that, with our present knowledge of the subject, he who resorts to a cutting operation in such circumstances is knowingly guilty of doing wrong.

The imaginary hiding-place of the last fragment, in the bladder, is the bugbear which deters many from attempting lithotripsy, and which is urged against this operation by its opponents. Those, however, who are at all experienced, give themselves no trouble about the last fragment, which, if too small to be felt, will surely escape with the urine. The assertion that, by lithotomy, stone is always entirely removed, is not true. There are many instances where fragments, and even entire stones, are left in the bladder after very carefully performed lithotomy. A patient is certainly no more exempt from the recurrence of stone after lithotomy than after lithotripsy.

The following cases are contributed to add to the already ample testimony in favor of lithotripsy where small stones are detected, and I am sure that few will object to the operation, as the introduction of a lithotriptor to seize and measure the stone is now practiced by good lithotomists; and the calculus, once in the jaws of the instrument, might as well be at once crushed by two or three turns of the wheel, and the patient as permanently relieved as by the more dangerous operation of lithotomy.

Case I.—Small uric acid calculus destroyed at one sitting; the detritus brought out partly in the jaws of the instrument—and partly by means of an evacuating character; the remainder expelled spontaneously the next day.

Thomas L., aged thirty-five (September, 1871.) History of nephritic colic obscure, but had been suffering from symptoms of stone for several months. When I first saw him, he had a very irritable bladder, and was not able to retain his urine—which was purulent, and at times bloody—for more than ten minutes. The patient underwent the usual preparatory treatment; the meatus urinarius, being too narrow, was freely incised. The first exploration (with Thompson's sound) revealed the presence of a stone emitting the charring characteristic of uric acid concretions. By the second exploration, made in a few days with a lithotriptor, the dimensions of the stone proved to be three-eighths by five-sixteenths of an inch.

On September 20th, 1871, the bladder having been rendered more tolerant by the treatment pursued, the operation was performed in presence of Prof. Gilmore, of Mobile, Prof. Westmoreland, of Atlanta, Georgia, and several other medical gentlemen. The calculus was reduced to a coarsely granular state by means of the English, plain-bladed lithotriptor, and the time occupied, including the introduction of the lithotriptor, the crushing of the concretion, and the withdrawal of the instrument, was three and a half minutes. An evacuating catheter was then introduced, and several small fragments and some dust were expelled. On the next day he passed more detritus, which was lost. There remained some vesical irritability, which was, however, soon removed by daily injections of warm water. I saw him in two, and again in eight months, and he was entirely well.

Case II.—Small uric acid calculus crushed at a single sitting; several grains of detritus removed with the lithotriptor—the remainder passed on the following morning.

Dr. ——, aged forty-eight (February 15, 1872.) Has had two attacks of nephritic colic, the last a year ago—the calculus coming from the left kidney. He had occasional attacks of hæmaturia, and his bladder was sometimes very irritable.

After a week's preparation, the stone was seized with a light lithotriptor and crushed at once (three minutes), and three grains removed in the blades of the instrument. Eight grains were expelled the next day, and the patient, contrary to advice, rode out during the afternoon, but suffered no inconvenience, and has ever since been free from trouble. The stone measured a quarter of an inch. Its composition was uric acid.

Remarks.—I believe the time will soon come when three operations of lithotripsy will be performed (in adults) to one of lithotomy—simply because surgeons will look for small stones, and find them, too, oftener than they have heretofore done. Very large concretions will be reserved for perineal lithotrity—the combination of median lithotomy with fragmentation of the stone—an excellent operation devised by Professor Dolbear, of Paris, and performed by him thirty times in the last ten years, with five deaths. I have lately operated, according to Dolbear's plan, on two cases where lithotripsy was contra-indicated by reason of the large size of the stones (one weighing two ounces, the other one ounce), and the existence of obstinate and troublesome cystitis. Both patients recovered.

